# The early-life gut microbiome in common pediatric diseases: roles and therapeutic implications

**DOI:** 10.3389/fnut.2025.1597206

**Published:** 2025-05-29

**Authors:** Taiwo Bankole, Yuanyuan Li

**Affiliations:** Department of Nutrition and Food Science, University of Maryland, College Park, MD, United States

**Keywords:** early childhood, microbiome, metabolome, pediatric diseases, gastrointestinal disorders, neurological dysfunctions, metabolic diseases, atopy

## Abstract

The early-life gut microbiome has been increasingly recognized as a contributing factor for pediatric health and diseases. Studies have reported that the human gut microbiota colonization commences at birth and progresses over the course of the first three years of life, until it reaches a mature and stable diversity and composition. During this critical window, the gut microbiome is vulnerably subjected to environmental factors, leading to transient microbial reprogramming and functional changes. The dynamic early-life intestinal microbiota is frequently manipulated by environmental factors, which impact the composition and function of the gut microflora, hence confer to short-and/or long-term health outcomes extending to adulthood. Evidence has shown that the imbalanced gut microbial community early in life is associated with several childhood diseases and disorders, such as inflammatory bowel diseases, allergies, attention-deficit/hyperactivity disorder and pediatric obesity. Manipulating the early-life intestinal microbes can either ameliorate or impair host’s immunological and metabolic responses, impacting overall health conditions later in life. This narrative review article discusses the recent understanding and implications of the early-life gut microbiome in common pediatric diseases and potential intervention approaches.

## 1 Introduction

The developing gut microbiota represents a comprehensive ecosystem that gradually transitions through a succession process during childhood toward a state of high diversity in an adult gut. The gut microbiome normally matures around the age of 3 years (1–4), but the maturation process can extend to about 6 years in humans (5). This period, referred to as “the critical window,” presents a vulnerable phase for the microbiota development during early life. A majority of the gut microbiome (60 to 70%) remains constant throughout the entire lifetime once the gut microbiota reaches an adult-like composition (6). During the early-life microbial development, the developing gut microbiome is more susceptible to a range of environmental factors. Consequently, this critical window creates a potential opportunity for microbiota reprogramming to recreate a protective gut ecosystem against various diseases later in life (1, 7, 8).

The gut microbiome normally undergoes 3 crucial developmental stages, including early developmental phase (3–14 months), transitional phase (15–30 months) and stable phase (31–46 months) (9). For years, the neonate’s gut has been considered sterile, only becomes colonized after birth transmitted from maternal microbiota, environment, and diet. However, many findings have reported that microbial exposure can retrospect as early as gestation stage *in utero* and early colonization occurs immediately after birth (2, 3). During the first 3 years of life, the gut microbiota undergoes dynamic progression with the predominant phyla colonization, including *Bacteroidetes*, *Firmicutes*, *Proteobacteria*, and *Actinobacteria*. The presence of *Bacteroidetes* shows a substantial increase from birth to 27 months and remains stable until 36 months. In contrast, *Proteobacteria* gradually decreases from birth to approximately 24 months. Phylum *Firmicutes* remains relatively stable throughout birth to 36-month period (10, 11).

The early-life intestinal microbiome is critically important for the development and function of the metabolic, immune, and neurological systems, required for healthy growth and development (2, 12, 13). Within a child’s early life, external influences may alter the interactions between the microbiota, host metabolism and immune systems, leading to either a pathophysiological or healthy state (1, 14). The developing gut microbiota plays a significant role in the biosynthesis of various metabolites, including bile acids, amino acids, short-chain fatty acids (SCFAs) and neurotransmitters (NTs). These metabolites gain access to systemic circulation and distal organs, to modulate various activities, such as inflammation and hormonal secretion (15, 16). The inner layer of the gut is made of epithelial cells that form a physical barrier between the colonic microflora and the underlying tissues, preventing the migration of detrimental bacteria and small molecules into the circulation. Tight junctions between the colonic epithelial cells function as gatekeepers that regulate the entry of nutrients and other substances into the intestinal epithelium. Gut barrier dysfunction has been reported to contribute to the onsets of many childhood diseases (17–19). In addition, the process of microbiome development has been identified as a crucial phase in the maturation and priming of a healthy immune system, and any disruption to this process may predispose to subsequent inflammatory or immune-mediated diseases (1, 20). As a result, the disrupted early-life gut microbiota can interact with different organ systems through various axes, contributing to the pathogenesis of various local and systemic pediatric diseases (17, 18).

The composition and function of the gut microbiome in early childhood has been increasingly implicated in the development of various common pediatric diseases (7, 10, 12, 21). Cumulative evidence has shown that dysbiosis of the early-life intestinal bacterial community and the subsequent dysregulated metabolites and immune regulations, are closely associated with the onset and progression of major childhood health conditions (1, 22). Interestingly, the plasticity of the gut microbiome early in life coincides with developmental processes in infants, suggesting that several pediatric diseases may have their gut developmental origin (1, 3, 22, 23). Thus, potential interventions or therapies targeting the gut microbiota during early life could serve as a new approach to potentially reverse the course of these dysbiosis-driven abnormalities. This narrative review article aims to explore the critical link between the early-life gut microbiota and dysbiosis-induced common pediatric diseases, with a special focus on childhood metabolic, neurodevelopmental, gastrointestinal (GI) and atopic diseases. Additionally, we discuss potential strategies for early-life microbial reorganization, to address how the gut microbiota during early life stage can be manipulated against common dysbiosis-associated pediatric diseases. In summary, this review provides pivotal evidence in the studies focused on the implication of the early-life gut microbiome development on childhood health and diseases because a foundation for a resilient and stable adult like bacterial communities is initiated during this critical developmental window. Literature search for this study was conducted in major scientific databases, including but not limited to PubMed and Google Scholar, through combination of keywords related to core concepts such as early life gut microbiome, specific pediatric disease, influencing factors and relevant mechanisms.

## 2 The early-life gut microbiome axes

**Figure 1:**
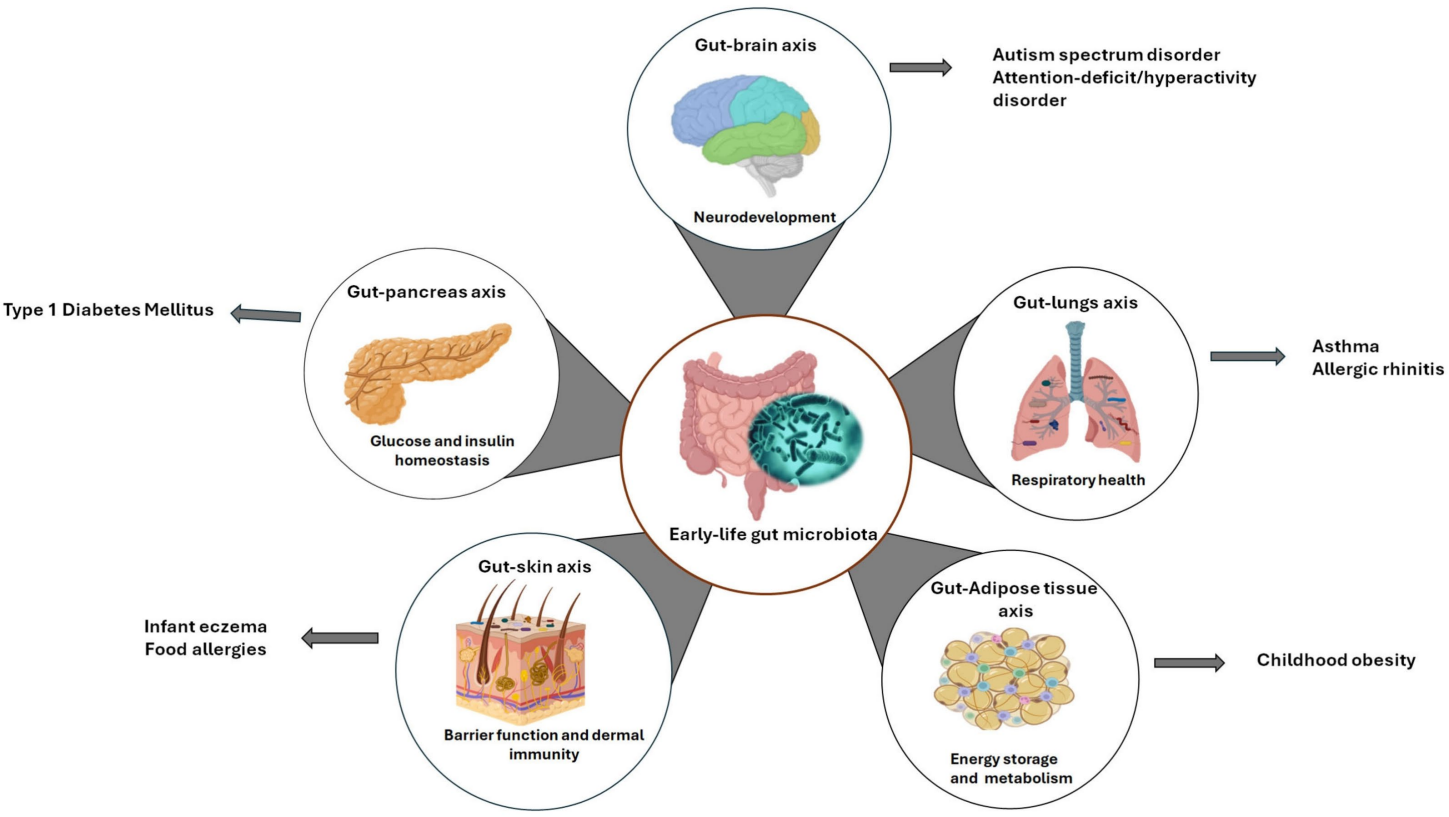
Early-life gut microbiota axes. This schematic depicts the bidirectional relationship between the developing gut microbiome and key organ systems related to common pediatric diseases. Created with BioRender.com.

The functions of the gut microbiome involve in modulating nutrient uptake, immune maturation, pathogen resistance and maintaining intestinal epithelial wall integrity, significantly contribute to the host-microbiota crosstalk. The early-life gut microflora can facilitate establishment of gut axes connecting to key distant organs such as brain, lungs, and skin, influencing their functions (Figure 1). Consequently, any disruption in the early-life microbiome during the critical developmental window can result in potential detrimental effects, leading to childhood diseases, and disorders (Figure 1) (24, 25).

The early-life gut microbiome participates in the development and maturation of endocrine, metabolic, and immune systems (13, 20). The gut microbes influence diverse physiological functions through their impact on host metabolism. Substrates such as dietary components, mucus, primary bile acids, can be metabolized by gut microbes and converted to small bioactive metabolites that modulate several key signaling pathways. For example, the microbiota-derived metabolic compounds can act as signaling molecules to regulate immune cells (16). The well-studied microbial metabolites are SCFAs, mainly including butyrate, acetate, and propionate, derived from dietary fiber fermentation in the gut (26, 27). Several studies have reported the roles of SCFAs in maintaining mucosal barrier integrity and regulating the host immune systems. Mechanistically, SCFAs can permeate cell membranes by passive diffusion, but are mostly absorbed by transporters expressed on intestinal epithelial cells such as proton-coupled monocarboxylate-transporter 1 and sodium-coupled monocarboxylate-transporter 1. Alternatively, SCFAs act as ligands for G-protein coupled receptors (GPCRs), including GPCR41, GPCR43 and GPCR109A, expressed on immune cells. In the lamina propria, innate, and adaptive immune cells produce cytokines and chemokines that regulate inflammatory processes (16, 26). SCFAs can epigenetically regulate gene expressions involved in epithelial tight junctions, as well as local and systemic immune cascades (26, 27). In children, low levels of SCFAs have been correlated with mucosa damage and intestinal wall hyperpermeability, known as leaky gut, which are linked to several childhood dysbiosis-associated diseases such as inflammatory bowel diseases (26). Notably, both gram-positive and gram-negative bacteria release membrane-bound extracellular vehicles that contain diverse bioactive molecules, including metabolites, nucleic acids, proteins, and lipids. Emerging evidence suggests that bacterial extracellular vesicles (BEVs) may cross intestinal epithelial barriers, gain access into the circulation, and disseminate to targeted organs to elicit specific metabolic, neurological, and immunological responses. This bacterial-host interaction also underscores the implication of BEVs in pediatric health and diseases (28–30).

During the first years of life, the brain and the gut microbiome develop simultaneously. Hence, the intricate interplay between the gut microbiota and neurological function occurs during early childhood. For instance, certain brain conditions have been linked to gut-related physiological consequences among children, indicating early-life microbial colonization may impact brain development (7, 24). The bidirectional signaling of the gut-brain axis involves multiple neurological systems, including the central nervous system (CNS), enteric nervous system (ENS), autonomic nervous system, hypothalamic–pituitary–adrenal (HPA), and immune and endocrine systems (23, 31, 32). Notably, the gut contains an extensive network of neurons known as the ENS, which functions autonomously. The vagus nerve that connects the ENS and CNS sends signals between the digestive system and internal organs to the brain, and vice versa (32). Signals generated by the hypothalamus reach the pituitary and adrenal glands and communicate with specialized enterocytes via the HPA axis (33). In addition, biochemical interactions within the gut-brain axis require a complex pathway involved in secreted NTs/hormones, cytokines, and bacterial metabolites. Intestinal microbes can directly synthesize neuroactive peptides (hormones and NTs) or stimulate their release from enteroendocrine cells through microbial metabolites. For instance, many microbial species such as *Lactobacillus*, *Bifidobacterium*, and *Bacteroides* have been reported to biosynthesize *γ*-aminobutyric acid (GABA) (28, 31, 33). Interestingly, it has been proposed that BEVs can transport gut bacteria-derived neuroactive molecules to the brain, eliciting neurological responses (28). NTs transmit signals to the brain via the vagus nerve and ENS, whereas hormones, cytokines, and SCFAs release into the bloodstream cross through the blood–brain barrier to alter cellular activity and eventually influence brain function. On the other hand, some neuroactive molecules secreted in the brain can disseminate to the gut to regulate GI functions (31–34). These findings imply that the brain and gut reciprocally influence each other.

Correspondingly, there is a bifurcate relationship between the microbiota in the gut and on the skin, referred to gut-skin axis. Through the gut-skin axis, the gut microbiota subsequently impacts the skin microbiome (17, 35). In early life, the developing gut microbiome influences skin health through its immunological and metabolic activities. Thus, skin diseases are most likely accompanied by alterations in the diversity and functions of the gut microflora, as well as the skin microbiome (25, 36). Certain enteric microbes and their metabolites influence cutaneous homeostasis by cross-talking with mucosal immune mediators and signaling cascades. Also, bacterial metabolites derived from the gut have been reported to enter the blood circulation to interact with skin receptors, consequently coordinating epidermal differentiation and keratinization, and modifying the skin’s bacterial compositions (25, 35, 37). For instance, skin exposed to ultraviolet B radiation from sunlight can subsequently initiate serum vitamin D production, which has been shown to associate with significant increment of the *α*-and *β* – diversity of the intestinal microbiota, suggesting the existence of the skin to gut relationship (38). More recently, the concept of gut-brain-skin axis has been reported, indicating the potential intricate interaction between the skin, brain, and gut (36, 39, 40).

In recent years, studies on the lung microbiome have gained significant advancements in understanding the gut-lung axis, an intricate pathway between the gut and respiratory system. Although the lung microbiota possesses a lower microbial biomass compared to the gut, studies show it significantly impacts immunity and homeostasis in the airway (41–43). Microbial composition and immune mediators of the gut and lung engage in a complex and interconnected crosstalk. The lung microbiota impacts gut immunity, contributing to immune tolerance via neutrophil recruitment, pro-inflammatory cytokines and antimicrobial peptides (AMPs). Also, enteric microbial metabolites can disseminate to the lung through blood circulation to regulate the pulmonary immune pathways. For example, SCFAs can act as signaling molecules on tissue-resident antigen presenting cells to alleviate allergic and pro-inflammatory responses in the lung. In response to inflammatory signals, innate lymphoid cells originating from the intestinal lamina propria have been found to migrate from the gut to the lung, contributing to tissue repair and host defense (41, 44, 45).

Research has suggested the existence of the gut-pancreas axis, which is a two-way communication between the gut microbiota and the pancreas via pancreatic ducts (46). Pancreatic tissue that is previously considered sterile has been increasingly shown to be colonized by several microflora both under normal and pathological conditions. Oral microorganisms may colonize the pancreas through the GI tract (47). It has been demonstrated that the Ca^2+^ channel Orai1 in the pancreatic acinar cells mediates the release of cathelicidin-related AMPs, which can regulate the gut microbiota and innate immunity, and barrier functions (47, 48). Alternatively, the gut microbiome modulates the secretion of pancreatic AMPs (46, 47). It has been suggested that microenvironmental factors can cross the intestinal barrier to the pancreatic islets to modulate inflammatory and autoimmunity milieus. Moreover, the gut microflora has been reported to migrate to the pancreas to influence pancreatic microenvironment (48, 49).

The gut bacteria can interact with adipose-tissue (AT) via the gut-adipose tissue axis, known as the microbiota-fat-signaling axis. The AT responses to the changes in the intestinal microbial-environment through the metabolite-driven pathways. Such microbial-derived metabolites can travel to the systemic circulation and act as signals on receptors that elicit certain responses in ATs, a mechanism known as the signal-receiver-response framework. For instance, SCFAs have been shown to inhibit lipolysis and promote adipocyte differentiation in ATs. Gut bacteria-derived lipopolysaccharides, when bound to toll-like receptors (TLR) can trigger inflammation and immune cells infiltration in ATs (50). On the other hand, AT can secrete certain hormones and cytokines to modulate the gut microbial composition and functions. These findings indicate that the interaction between the gut microbiota and adipose tissue is bidirectional (19).

Taken together, the gut microbiome engages a bidirectional crosstalk with distant organs through specialized axes, critically influencing the pathogenesis of childhood diseases. These axes spanning the gut-brain, gut-AT, gut-pancreas, gut-lung, and gut-skin interfaces exert systemic effects via immune, metabolic and neuroendocrine pathways. Mechanistically, the altered gut microflora profiles can disrupt these interactions and initiate pathogenesis through aberrant microbial metabolite signaling, barrier dysfunction and immune dysregulation (17, 50). For instance, the early-life gut dysbiosis disrupts the gut-brain axis that controls neurodevelopment and behavior, contributing to autism spectrum disorder (ASD) and attention-deficit/hyperactivity disorder (ADHD) through defective NTs synthesis and neuroinflammatory cascades (51, 52). Dysfunctional gut-AT axis is observed in childhood obesity, characterized by white adipose tissue inflammation through TLR activation by gut-derived ligands, and dysregulated SCFA-mediated lipolysis inhibition (50). In type 1 diabetes mellitus (T1DM), early-life microbiota disturbances in the gut-pancreas axis may provoke *β*-cell autoimmunity via loss of mucosal immune tolerance and molecular mimicry (53). Moreover, alterations in the gut-lung axis heighten susceptibility to asthma and allergies. Gut microflora imbalance contributes to asthma through bacterial structural components and metabolites, such as lipopolysaccharides and peptidoglycan, triggering dysbiosis-induced T-helper (Th)-2 skewing and airway inflammation. Asthma, in turn, may cause inflammation-induced intestinal damage (17, 54). Additionally, dysfunctions in the gut-skin axis exacerbate infant eczema because pathogenic interactions and inflammatory signaling in the gut may compromise barrier integrity, precipitate immune reactions and disrupt skin immune homeostasis (17, 55). In common childhood GI disorders such as inflammatory bowel diseases (IBD) and irritable bowel syndrome (IBS), altered gut microbiota profiles and metabolite production, as well as a compromised mucosal barrier result to GI-specific inflammation and intestinal damage (56). Collectively, these axes underscore the roles of the gut microbiome as a central modulator of interorgan communication, with its dysregulation during critical developmental windows predisposing children to multisystemic diseases.

## 3 Manipulating the early-life gut microbiome

**Figure 2:**
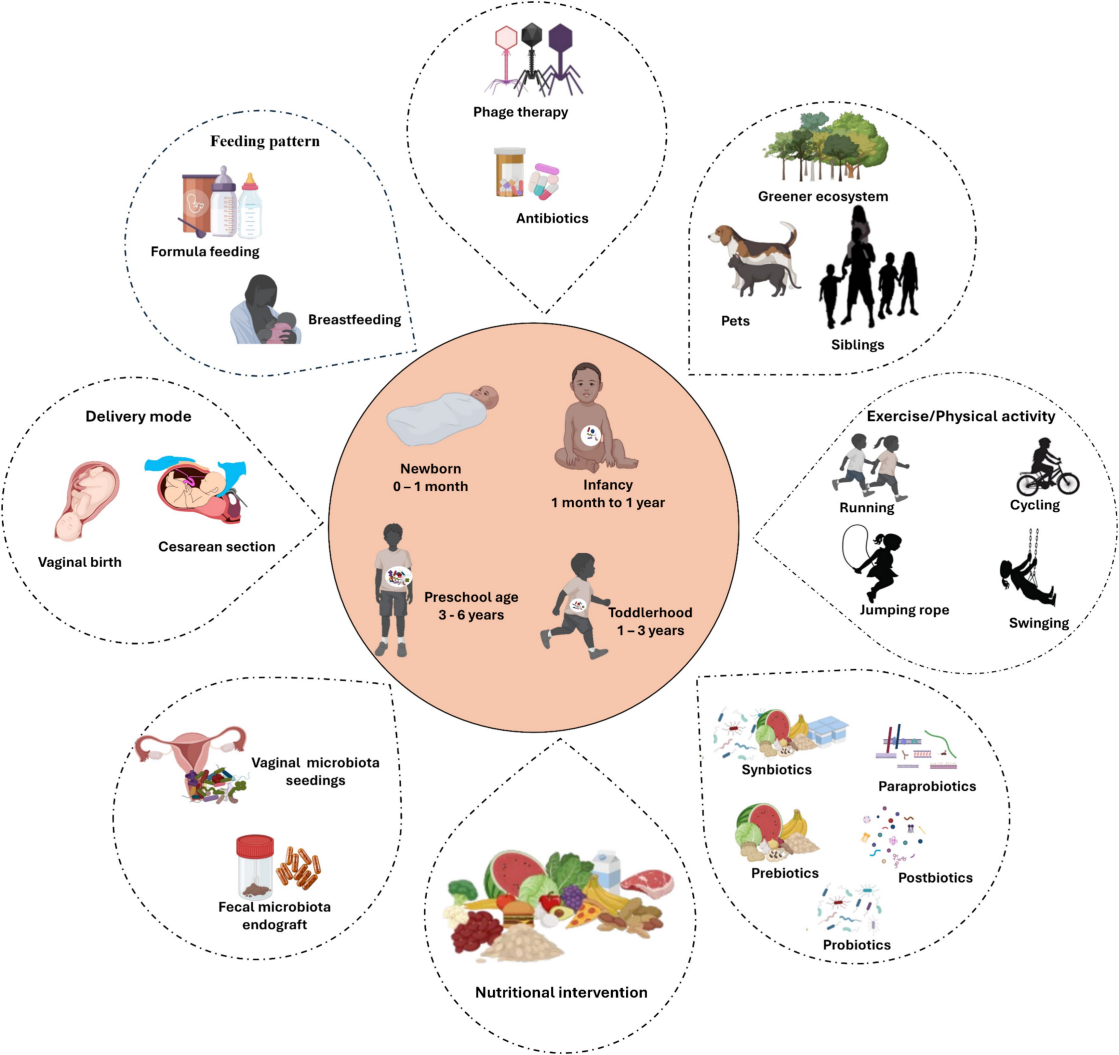
Factors that manipulate microbial development during early lifetime. Illustration underscores key external factors influencing the gut microbiome development and composition during early life stages, from birth to the first few years of life. The central hub depicts the major microbial developmental stages. Created with BioRender.com.

The development of the gut microbiome during the first few years of life significantly impacts a child’s health. Therefore, manipulating the childhood gut microbiota is considered as a promising strategy to combat the increasing prevalence of pediatric diseases. Understanding the factors that influence the development of the early-life gut microbiome provide promising opportunities for establishing a healthier and resilient gut microbiome in infancy and early childhood stages (Figure 2).

### 3.1 Delivery methods

The main delivery methods of childbirth, vaginal delivery and cesarean section (CS), remain the foremost contributing factor that significantly influences the composition and development of the gut microbiome. In a conventional delivery, diverse bacterial strains from maternal fecal and vaginal inoculum, such as *Lactobacillus* and *Bifidobacterium*, are exposed to the neonates (4, 12). As opposed to vaginal delivered babies, CS babies have shown delayed colonization of *Bifidobacterium* and *Lactobacillus*, low diversity of *Bacteroidetes*, but predominantly the genus *Enterococcus*, which are mostly found in hospital environment (2, 10, 57). Several studies have investigated the variations in microbial composition and stability associated with delivery methods. Infants delivered through vaginal births exhibit higher microbial stability and diversity, with similar fecal microbiota composition to their mothers, suggesting the important roles of maternal microbial exposure during vaginal delivery (15). On the contrary, CS infants have been associated with distinct alterations in gut microbial composition with lower *α*-diversity and similar to maternal skin and oral cavity (10, 15). While the gut microbiota develops gradually toward an adult-like profile, significant disparities in microbial communities between CS and vaginal babies are observed during the early lifetime, but tend to diminish over time (58). Importantly, studies have reported that the gut microbial variances observed in CS infants may be associated with increased risk of non-communicable diseases, such as asthma, T1DM and obesity, that appear early or later in life (12, 21). However, many of these studies have shown inconsistent results (59).

In opposition to the sterile womb theory, bacterial colonization begins during *in-utero* fetal life (12). Literatures have reported the presence of non-pathogenic bacteria in the placenta, amniotic fluid, umbilical cord, fetal tissues and meconium, translocated from the mother’s gut to the fetus during gestation. These microbial communities in the intrauterine environment can also be influenced by the changes in maternal microbiota caused by maternal health conditions and dietary patterns, as well as pregnancy-induced microbial variations, which are considered the decisive influence on infant’s early-life gut microbiota (12, 27).

In addition, maternal vaginal and fecal microbes represent the foremost and vast sources of bacteria exposed to infants (13, 60). Studies have investigated the effects of maternal vaginal seedings on the early-life gut microbiome, which refers to a process by applying a mother’s vaginal fluids to a CS newborn. Studies have shown that maternal vaginal seeding can partially restore infant’s microbiota colonization and increase mother to newborn microbial transfer (61, 62). On the opposite, other studies indicate that maternal vaginal seeding does not affect the early-life gut microbiota development (60, 63, 64). Discrepancies in results may be due to the fact that maternal vaginal bacteria do not persistently colonize the infant’s gut (13, 63). Because maternal GI tract has high microbial diversity, fecal microflora that colonize the infant gut may have long-term impacts. In respect to this, engraftment of maternal fecal microbes has been demonstrated to fully restore vertical microbial transmission from mother to child, thereby restructuring the early gut microbiota of neonates (58, 63, 65). Due to this remarkable outcome, maternal fecal transplantation is considered more effective in reshaping the infant microbiome compared to vaginal seeding (63, 65). Moreover, fecal microbiota transplantation (FMT) from a healthy donor has been reported to rebalance the gut microbial communities in children, leading to healthy clinical outcomes. However, the use of FMT in common pediatric diseases is still limited and preliminary (66, 67).

### 3.2 Feeding patterns

Human breast milk (HBM) not only provides sufficient nutrition for infant development but also plays a critical role in maturation of the gut microbiome during early life (68). HBM contains many bioactive components, such as immune cells, cytokines, lactoferrin, antimicrobial proteins and peptides, antibodies and human milk oligosaccharides (HMOs), that can positively modulate the infant’s gut microbiota (21, 58). In contrast to cow’s milk, HBM contains more than 250 various types of HMOs essential for modulating infant’s intestinal microbes and impacting immune system (4, 69). Over the past years, studies have demonstrated significant microbial changes in breastfed infants compared with formula-fed infants (15, 70). Breastfed infants typically have *bifidobacteria*-dominated microbiota, whereas formula-fed infants have a more diverse gut microbiota (2, 8, 70). Due to the overall health benefits linked to breastfeeding, the World Health Organization (WHO) recommends early introduction of breastfeeding within one hour of birth, exclusive breastfeeding for the first 6 months and continuous breastfeeding through at least the first year of life, and longer if desired (1, 69, 71).

Despite the well-appreciated practices of breastfeeding, many mothers are facing challenges that hinder breastfeeding abilities, such as mastitis, insufficient milk supply and postpartum depression (71). Thus, recent commercial infant formula manufacturers start to incorporate synthesized HMOs, such as 2́ -fucosyllactose and lacto-N-neotetraose, that are found in HBM with the potential of promoting the development of a healthy early-life gut microbiome (4, 69). For instance, a recent study revealed that infant formula fortified with HMOs shifted the gut microbial composition to resemble that of their breastfed counterparts with the abundance of *Bifidobacterium infantis* and reduced presence of toxic *Clostridioides difficle* (72). Moreover, infant formulas with HMOs can boost intestinal immune system development, as well as overall health and growth outcomes (72, 73).

Mechanistically, it is believed that microbes in HBM can be vertically transmitted from mother’s gut to the child through the entero-mammary pathway, which requires the uptake of gut bacterial strains by lymphocytes, such as dendritic cells (DCs). DCs can penetrate the intestinal epithelium reaching the mammary glands via the lymphatic circulation (68). Similarly, it has been hypothesized that maternal derived BEVs present in HBM can potentially contribute to the infant gut microbial colonization. As aforementioned, BEVs that are secreted from maternal gut bacteria and transmitted to the mammary glands, consist of bioactive components including microbial-associated molecular patterns. Upon ingestion by the infant during breastfeeding, BEVs interact with the intestinal epithelium and immune cells, thereby influencing the composition and function of the infant gut microbiota (29, 30). Altogether, these findings imply that the state of the maternal gut bacteria can influence the diversity and composition in HBM, subsequently influencing the infant gut microbiome during early life.

### 3.3 Nutritional interventions

Nutrition plays a pivotal role in influencing health outcomes through, at least in part, reshaping the gut microbiome during early life. Therefore, understanding how dietary macro-and micro-nutrients contribute to the priming and modulation of early-life gut microbiota is of paramount importance (74). Overnutrition, including maternal overweight and obesity, mostly caused by western dietary patterns or excess calorie intake, can induce an imbalanced gut microbial community, which is characterized by diminished microbial diversity linked to enhanced energy harvest and inflammation. These dysbiotic bacterial patterns can be vertically transferred from mothers to offspring during gestation, delivery or lactation, resulting to compromised infant gut colonization and immune tolerance, and increased risk of later-life diseases. Thus, healthy maternal nutrition offers a promising strategy to ameliorate the early-life gut microbiome, leading to improved health outcomes in the offspring (75, 76). On the other hand, early childhood malnutrition can also elicit gut dysbiosis, leading to increased presence of pathogenic microbial communities, such as *Gammaproteobacteria* (17, 77).

Complementary feeding after 6 months of life contributes to the transition of infant’s gut from a *bifidobacterium*-dominating community toward a more diverse microbial community, which reflects the introduction of more complex dietary patterns (8). Importantly, timing and types of infant diets are also vital for microbial development. It has been reported that early or delayed introduction of solid foods negatively alters the initial stages of microbial maturation and composition. As a result, it is recommended to provide infants with nutrient-dense complementary foods starting from 6 months of age (8, 78). A recent study by Mokhtari et al. indicates that the consumption of dietary fiber derived from various whole grains, legumes, fruits, and vegetables during infant’s complementary feeding stage led to a significant abundance of butyrate-producing bacterial species, such as *Faecalibacterium*, *Coprococcus*, *Dorea*, and *Oscillospira* (79).

Several studies have provided evidence on the impacts of different macronutrients on the diversity and composition of the gut microbiota, as well as metabolites derived from microbial activity (8, 57). For instance, high fiber diets (a preferred energy source for key gut microbes in producing beneficial SCFAs) and low glycemic-index foods help to maintain an eubiotic state by stimulating the growth of healthy bacteria in gut, such as *Prevotella*, *Bifidobacterium*, *Lachnoclostridium* and *Roseburia*. Conversely, high glycemic index and western diets have been demonstrated to shift the gut microbes toward a dysbiotic intestinal environment that favors the growth of harmful microbes (80–83). Dietary proteins are important sources of amino acids required for fundamental growth and development. Compared to animal proteins, plant proteins, such as lentils and legumes, are healthier for the growth of colonic bacteria. Plant-based diets are typically rich in dietary fiber and contain adequate quantities of polyunsaturated fatty acids that are beneficial to the gut microbiome. Regardless, consuming appropriate amounts of both animal and plant proteins is highly recommended to maintain gut homeostasis (84–86). Despite advancements in dietary interventions and the intestinal microbiota, the mechanistic interplay between macronutrients and the early development and modulation of gut microflora remains largely underexplored.

Micronutrients, such as vitamins, minerals, and phytonutrients, have been demonstrated to regulate the early-life gut microbial signatures. Several studies in animals, human, and cell culture have revealed the effects of both fat-soluble and water-soluble vitamins in modulating the gut microbiome and metabolomics profiles. For instance, vitamins A, B2, D, E, and *β*-carotene increase the abundance of beneficial commensals. Vitamins B2 and E can increase beneficial SCFAs producing-bacteria. Notably, intestinal microbes are also considered as vitamin synthesizers (87–89). A growing number of studies has investigated the regulatory impacts of various vitamins on the gut microbiota composition during the early years of life. Of these, majority indicate that vitamins promote the growth of beneficial microbes such as *Bifidobacterium* and *Akkermansia*, and inhibit illness-causing bacteria such as *C. difficile*, contributing to maintaining intestinal microbial homeostasis (90–92).

Additionally, minerals have emerged as potential modulators for the gut microbiota. For example, supplementation of magnesium (93), calcium (94), and selenium (95, 96) has been associated with increased overall diversity and beneficial colonic microbes. Conversely, iron supplementation in infants may have a negative impact on the composition of the gut microbiome, leading to a reduction in beneficial species like *Bifidobacterium* and *Lactobacillaceae* spp., and an increase in pathogenic species (97–99). However, it is important to note that research on the mechanistic roles of essential minerals in shaping the early-life gut microbiome is still not fully understood.

Recently, studies on the impacts of phytonutrients, specifically polyphenols, on the modulation of the early-life gut microbiome have received significant attention. Polyphenols, including flavonoids, carotenoids, and stilbenes, are bioactive compounds abundant in fruits, vegetables, herbs and microgreens. They possess strong antioxidant, anti-inflammatory and antimicrobial properties, contributing to disease prevention and overall well-being (100, 101). In addition, polyphenols play important roles in the regulation of the host microbiome profile. For example, grape polyphenol extract administered during early life can enhance the growth of beneficial probiotic bacteria such as *Akkermansia* and *Lactobacillus* (102, 103). Likewise, maternal exposure to dietary polyphenols during pregnancy and lactation may help reshape the offspring early-life gut microbiome. Our previous study showed that maternal soybean genistein diet can favorably shift the offsprings’ gut microbiota profiles to a more resilient state, contributing to an improved metabolic health and low risk of breast cancer later in life (104, 105). For instance, enriched abundance of genera *Bifidobacterium* and *Allobaculum* were found in progenies whose mothers were fed genistein-rich soy diet (104). Another study by Wei et al. indicated that maternal diet of sulforaphane glucosinolate (SGS) influenced the early life gut microbiota in the offspring (106). SGS was found to significantly increase microbial diversities and the abundance of *Prevotellaceae* and *Ruminococcus 1* in the treated offspring compared to controls.

Collectively, these findings indicate both dietary macronutrients and micronutrients have potential to modulate the early-life gut microbiome. Appropriate nutrition intervention during this critical developmental stage and sensitive window can facilitate achieving optimal healthy outcomes through reshaping the early-life gut microbiome. Therefore, nutrient intakes should strictly adhere to the recommended dietary guidelines for pregnant and lactating mothers as well as infants and young children, supporting the gut health in prevention of childhood diseases during early lifetime.

### 3.4 The roles of prebiotics, probiotics, synbiotics, postbiotics, and para-probiotics

The introduction of prebiotics, probiotics, synbiotics, postbiotics, and para-probiotics in early childhood has garnered attention as potential strategies for supporting a healthy gut microbiome and prevention of childhood diseases (71, 107). Prebiotics, also known as intestinal fertilizers, are dietary fibers of non-digestible carbohydrates. Fermentation of prebiotics in the gut lumen supports the growth of beneficial bacteria and facilitates the release of bioactive metabolic compounds that benefit the host (108). For instance, fermentation of galacto-oligosaccharides induces proliferation of microbial species such as *Veillonella*, *Bifidobacteria*, and *Lactobacilli* spp., and facilitates the release of SCFAs as microbial-derived beneficial metabolites (108, 109). Also, HMOs in breastmilk and most baby formulas are a great source of prebiotics in early life that support the growth of healthy enteric bacteria and induce overall protective effects (71). Several studies found that infants supplemented with prebiotics have increased microbial diversity with significant levels of beneficial microbes such as *Bifidobacterium* and *Lactobacillus*, and reduced pathogenic microbes (107, 110), while others have reported no such changes (110, 111).

Probiotics such as *Bifidobacterium and Lactobacillus* species, classified as live and non-pathogenic gut microbes, have been well-reported to confer optimal health to the host while administered adequately. Probiotics can be administered as supplements for a direct microbial introduction to the gut (21, 112). HBM is enriched with probiotic strains, making it a natural probiotic resource for early infancy (68, 113). Numerous studies have demonstrated the health benefits of probiotics supplementation in modulating early-life gut microbes (21, 112, 114). Fecal microbial reprogramming is partly attributed to the ability of probiotics colonization to enhance the diversity of beneficial colonic microbes and metabolites, contributing to reduced risks of childhood diseases through correction of early-life gut dysbiosis (112, 115).

Synbiotics, a combination of both prebiotics and probiotics, follows a synergistic or complementary approach of shifting the gut microbiota composition to a healthy state. Importantly, prebiotic components in the synbiotic formulation enhance the growth of probiotic microbes in the gut (112). Because of high amount of the prebiotic and probiotic components in HBM, it is considered a natural synbiotic for early-life gut microbiota development (116). Synbiotic supplementation has been demonstrated to increase bacterial diversity as well as improve gut health and resilience in early childhood (43, 117, 118).

In recent years, postbiotics have surfaced as a therapeutic approach and are known to confer profound health benefits to the host (18, 119). Postbiotics are bioactive compounds derived from beneficial bacterial metabolism. Major sources of postbiotics include HBM, fermented foods, products from probiotic metabolic processes (e.g., SCFAs, peptides, vitamins, enzymes etc.) and BEVs (113, 120, 121). Evidence has been recognized that nutritional intervention of postbiotics can promote the establishment of enteric beneficial microbes and augment a positive influence on pediatrics health (18, 120). Within the early pediatric age group, studies have investigated the effects of postbiotics as potential modulators on GI environment. Postbiotics have been reported to increase the production of SCFAs and its producers, as well as establish colonization resistance against disease-causing microbes (120, 121).

On the other hand, para-probiotics such as peptidoglycans, cell-surface proteins, and cell-wall polysaccharides, are defined as non-viable microbial cells of probiotics that provide health benefits to the host when administered adequately (122, 123). Similar to probiotics and postbiotics, the use of para-probiotics to induce positive clinical outcomes has been applied in the pediatric population (119). However, more studies are warranted to explore the effects of both para-probiotics and postbiotics on the early-life gut microbiome, and the mechanisms underlying how they influence early disease pathogenesis.

### 3.5 Exercise

Exercise or physical activity (PA) regulates energy production, energy expenditure and food intake through the gut microbiota composition. Emerging research has demonstrated that PA plays a crucial role in shaping enteric microflora by increasing the beneficial microbial species, microbial diversity and SCFAs producers. Hence, promoting PA among children is important for gut health (124, 125). Metagenomic analyses have shown positive association between the abundance of *Roseburia*, *Akkermansia* and PA (126, 127). In contrast, sedentary lifestyle and lack of PA affect the diversity, composition and functions of the gut microbiota. Studies have shown that sedentarism alters microbial diversity with increased abundance of energy harvesting microbes such as *Firmicutes*, and low abundance of *Actinobacteria* (125, 128).

An animal study conducted by Mika et al. (129) investigated whether exercise (in form of wheel running) during juvenile period will produce more microbial changes in comparison with adult exercise. This study indicates that, as opposed to adult runners and sedentary juveniles, early-life juvenile exercise can significantly alter several phyla (e.g., increased *Bacteroidetes* and reduced *Firmicutes*) and genera that are associated with increased lean body mass. While the intervention of PA on early-childhood gut microbiome is still underexplored, it is reasonable to predict that habitual PA may have potential benefits for the development of a healthy gut microbiota from early lifetime. WHO recommends that toddlers and preschoolers (under 5 years) should spend at least 180 min per day on various PAs to achieve optimal health benefits (130, 131). Particularly, a well-balanced diet and regular PA have been shown to increase the abundance of favorable colonic microbes. Thus, nutritional status in addition to PA can strengthen the positive influence on the gut microbiota development in early childhood (132, 133).

Prenatal and antenatal PA also show impacts on the offspring gut microbiome. For instance, maternal exercise, before and during pregnancy, has been reported to induce remarkable changes in the gut microbial composition of the offspring by reducing detrimental bacteria and enriching SCFAs-producers accompanied by improved overall metabolic health (134, 135). Also, it has been speculated that changes in infant’s early gut microbiota may be due to the HMOs changes in breastmilk, induced by maternal exercise (136). However, further studies are required to reach a definite conclusion.

### 3.6 Antibiotics exposure and phage therapy

Antibiotics treatments are common practices against various infectious diseases. However, the overuse of antibiotics during early childhood has been associated with a myriad of short and long-lasting negative consequences, including antibiotic-resistance health conditions (137, 138). Also, the abuse of antibiotic treatment in children has been shown to negatively impact the gut microbiota composition, leading to gut dysbiosis and the associated health outcomes, such as obesity and asthma (14, 139). In recent years, several studies have highlighted the effects of antibiotics overuse on the early-life gut microbiome, resulting from maternal antibiotic intake during pregnancy and lactation, as well as direct antibiotic exposure to infants (140, 141). Intrapartum antibiotic administration has been suggested to remodify neonatal gut microbes through antibiotics passage via the umbilical cord to the fetus and alterations in maternal vaginal and fecal bacteria (140). Particularly, reduced abundance of overall microbial biodiversity and beneficial microflora such as phyla *Actinobacteria* and *Bacteroidetes,* as well as predominance of potentially pathogenic microorganisms such as *Proteobacteria*, were observed in infant fecal microbiota after maternal exposure to antibiotics during labor (11, 142, 143). In addition, intrapartum antibiotic exposure has been found to alter HBM microbes, which directly impede the development of beneficial and healthy bacterial strains, such as *bifidobacteria* and *lactobacilli*, to colonize the infant gut (141, 144). Similarly, direct postnatal exposure to antibiotics in children has been demonstrated to shift the gut microbiota composition to a depleted *Bifidobacterium* population, and elevated abundance of *Enterococcus* and *Klebsiella* (145, 146).

The surge of infectious diseases has prompted the use of phage therapy (147). Bacteriophages (phages) possess bactericidal mechanisms that selectively target pathobionts and antimicrobial-resistant bacterial strains. Thus, phages play an important role in changing the gut microflora composition, diversity, and function (148, 149). Recent findings have reported that phages can recover the intestinal microbiome homeostasis by restoring the microbiome’s balance and stability (148, 150). Among the pediatric population, phage therapy has been proposed as a treatment strategy to combat multidrug-resistant bacterial infections and pathologies, such as bacteremia and chronic lung infections (151). However, the influence of phage therapy on the development of gut microbiota composition in young children remains largely understudied.

### 3.7 Environments

In early life, the development of the gut microbiome is significantly influenced by various aspects of the social and physical environments. Factors such as daycare attendance, hygiene practices, caregiving, home environment, pets and presence of siblings play crucial roles in shaping the early-life gut microbiome. Consequently, any disruption in these environmental factors may have notable impacts on the early-life gut microbiome (151, 152). Interestingly, the onset of coronavirus disease 2019 (COVID-19) pandemic has been shown to induce profound changes in gut microbiota of children. During the COVID-19 pandemic, the infants’ fecal samples have shown a lower microbial diversity than the counterparts before the pandemic. These changes may be attributed to decreased social interactions and increased usage of detergents, disinfectants, and hand sanitizers (153, 154). Also, studies have indicated that prenatal and/or postnatal contacts with household pets and siblings have a significant impact on the early-life gut microbiome. In these studies, infants living with pets are shown to associate with increased bacterial richness and diversity, and enriched abundance of *Oscillospira* and *Ruminococcus* in their guts (152, 155). Likewise, children living with older siblings are positively associated with increased bacterial diversity and richness (156, 157). Importantly, early-life contacts with pets and siblings increase infants to surrounding microbes such as soil/outdoor microbes and human/animal microbiota, which promote gut microbial changes during early childhood (152). Furthermore, a recent study showed that increased maternal exposure to greener environments in residential areas elevates breastmilk’s HMOs diversity and concentrations, which can subsequently influence the infant’s gut microbiome (158, 159).

## 4 Early-life gut microbiome in common pediatric diseases

The development of the early-life gut microbiome has a long-lasting impact on human health (10). Since the period from birth to 3–6 years of age is regarded as the critical window for the development of the gut microbiome, any disruptions during this period may jeopardize the early-life gut microbiome development and subsequently increase the risks of childhood disease and disorders (1).

**Figure 3:**
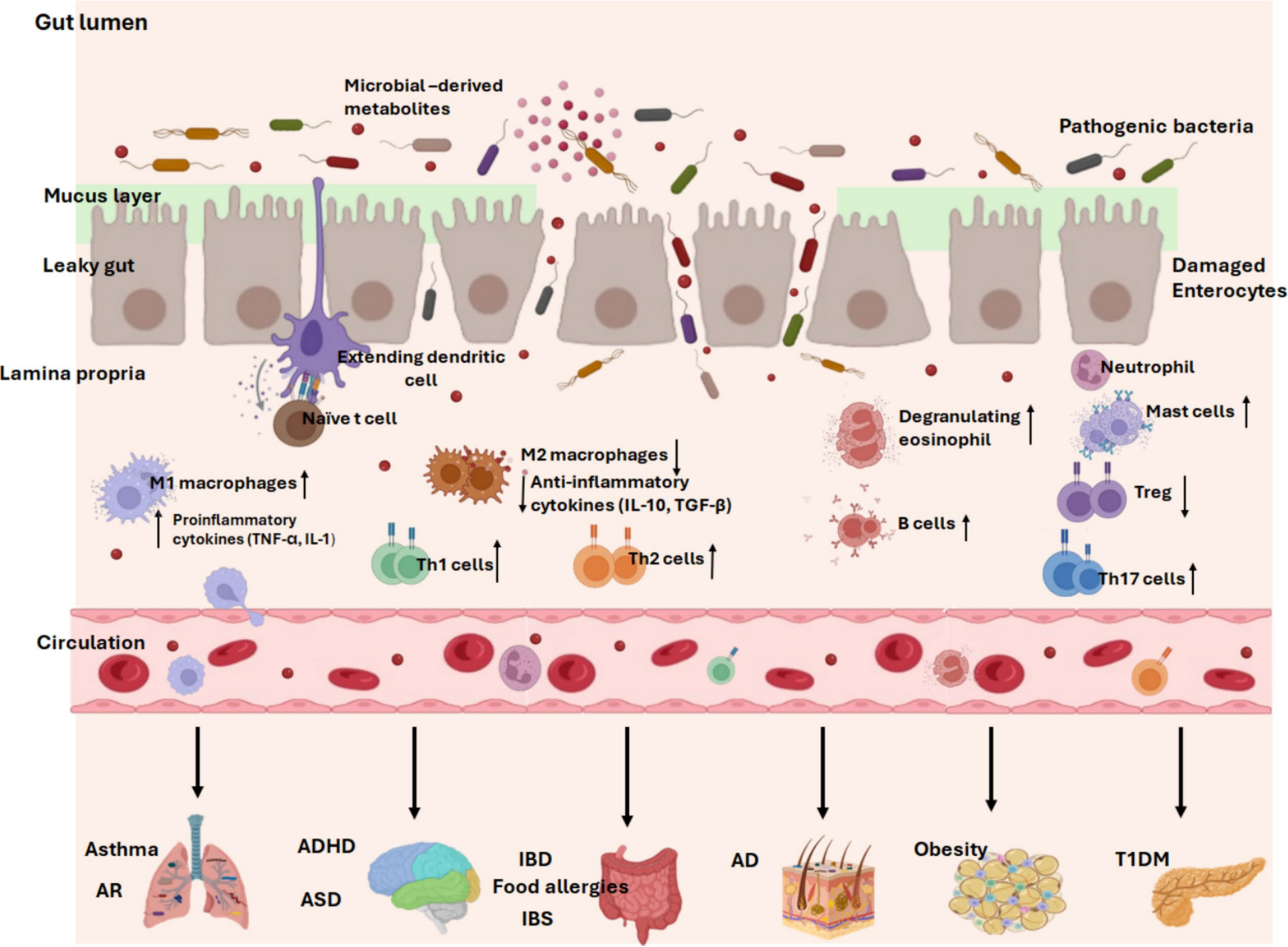
Early-life gut dysbiosis in common pediatric diseases. Dysbiosis of the gut microbiota alters the balance of immune function. With a compromised intestinal barrier, pathogenic bacteria invade the intestine and trigger the release of multiple pro-inflammatory cytokines from M1 macrophages (TNF-*α*, IL-1), skew naïve T cell differentiation into Th1, Th2, or Th17 cells, increase immunoglobulin-producing B cells, degranulate eosinophils and mast cells, and reduce anti-inflammatory mediators from M2 macrophages (IL-10, TGF-*β*). Long-term response of the immune system to pro-inflammatory responses eventually leads to the development of inflammatory diseases. In addition, a leaky gut facilitates the entry of harmful microbiota-derived products and pro-inflammatory mediators into the systemic circulation, where they are transported to distant tissues and trigger inflammation in multiple sites, contributing to childhood diseases. AR, allergic rhinitis. ADHD, attention-deficit/hyperactivity disorder. ASD, autism spectrum disorder. T1DM, Type 1 diabetes mellitus. IBD, inflammatory bowel diseases. IBS, irritable bowel syndrome. AD, atopic dermatitis/eczema. Created with BioRender.com.

It is established that the gut microbiota and mucosal immune system engage in extensive bidirectional communication. The early-life microbiota colonization plays critical roles in the maturation and education of the host’s innate and adaptive immune responses. Postnatal period and early infancy also represent a crucial window for the establishment of a mature and beneficial microbiota, and priming period for immune cell maturation and homeostasis. Of note, immature infant immune system is linked to increased susceptibility of childhood diseases (160–162). Mechanistically, the gut microbiota establishes a dynamic ecosystem that interacts with the host through the production of microbial-derived ligands and metabolites, which can not only directly influence the maturation and function of immune cells in the gut-associated lymphoid tissue but can also reach distant tissues via systemic circulation to modulate immunity (160, 163). For instance, SCFAs can regulate intestinal barrier function and promote the differentiation of regulatory T cells (Tregs) and monocyte-to-macrophages to foster immune tolerance and reduce the risk of allergic and autoimmune diseases (160, 163). Therefore, the intestinal microbiota and their metabolites are critical mediators for the early development of host immune system that determines the pediatric health outcomes (Figure 3).

Both the innate and adaptive immune systems have been shown to regulate the composition and function of the gut microbiota (161). For instance, intestinal AMPs produced from specialized secretory Paneth cells, are considered as an ancient type of innate immunity. AMPs selectively target detrimental pathogens through direct antimicrobial activity, thus maintaining a healthy balance of gut bacteria (160). Also, the TLR5 gene expressed on intestinal epithelial is critical in shaping microbiome composition early in life. Lack of TLR5 expression in neonatal mice has been reported to alter the gut microbiota that can persist until adulthood, resulting in increased risk of chronic diseases (163). HBM, a rich source of maternal antibodies, predominantly secretory IgA, provides the first source of adaptive immunity in the neonate’s intestinal tract and promotes the development of healthy microbiota that provides long-term protection (164, 165). Foxp3^+^ Treg cells localized in Peyer’s patches also promote production of secretory IgA from class-switched B cells, which contributes to homeostatic gut microbiota (160).

Importantly, early-life gut microbial dysbiosis is recognized as a key determinant of immune dysregulation, driving mucosal inflammation through aberrant activation of multiple immune cell populations (162). A study by Henrick et al. demonstrated that early-life dysbiosis, characterized by a lack of *bifidobacterial* spp. and HMO-utilization capacity, was linked to systemic inflammation and immune dysregulation (166). Supplementation with *Bifidobacterium infantis* that can facilitate utilization of HMOs appeared to modulate immune cell populations, specifically by silencing pro-inflammatory Th2 and Th17 responses, inducing IFN-*β*, and upregulating immunoregulatory galectin-1 in Th2 and Th17 cells. This suggests that *Bifidobacteria infantis* may promote a healthier and more balanced immune state. Additionally, the gut microbiota’s influence extends beyond the immune system, impacting tissue specific developmental functions via the gut axes (Figure 3). As such, disruptions in the immune-microbiota interactions during critical developmental windows may predispose children to a variety of childhood conditions such as neurodevelopmental disorders, metabolic conditions, allergies and inflammatory diseases (160, 161). A study carried out in infants with allergic diseases showed increased fecal abundance of *Ruminococcus gnavus*, which has been reported to stimulate secretion of epithelium-derived cytokines and further activate ILC-2 and DCs to promote differentiation of Th2 cells and production of their cytokines. This resulted in infiltration of colon and lung parenchyma, leading to onset of allergic responses (167). Since gut dysbiosis significantly influences the progression of pediatric diseases, changes in the functional readout of microbial activity such as microbial metabolites and immunological signatures may implicate the susceptibility of common childhood diseases (168) (Table 1). Taxonomic abundances of the gut microflora and metabolomic signatures associated with common pediatric diseases are summarized in Table 1.

**Table 1:** Modifiable factors and early gut bacteria-metabolomic dynamics observed in children with/at elevated risk of common pediatric disorders.

Pediatric diseases	Gut microbiota (enriched)	Gut microbiota (depleted)	Metabolomics changes	Manipulating factor
Allergic conditions				
Atopy (common to all 4 allergies)	*Ruminococcus gnavus* (167) *Eggerthella lenta*, *Escherichia coli*, *Enterococcus faecalis*, *Clostridium innocuum*, and *Tyzzerella nexilis* (172)	*Anaerostipes hadrus, Fusicatenibacter saccharivorans*, *Eubacterium hallii*, and *Blautia wexlerae* (172).	↓ Fecal butyrate, acetate ↑ Tryptamine, phenylethylamine, tyramine (172)	Positive factor: Breastfeeding (199, 204) Early-life fiber diet (204) Postbiotics (204)
Infant eczema or AD	*Enterococcus* spp., *Peptostreptococcaceae*, *Eggerthella*, *Coprobacillus*, *Peptoniphilus* spp., *Bacteroides* (254) *E. coli*, *Klebsiella pneumoniae* (255)	*Sutterella* spp., *Fusobacterium* spp. (254) *Bacteroides fragilis*, *Erysipelotrichaceae*, *Eubacteriaceae*, *Lachnispiraceae*, *Ruminococcaceae* (255).	↑ Fecal glucose concentration ↓ Fecal butyrate, acetate, propionate (255)	Positive factor: Probiotics (197) Synbiotics (198) Negative factor: Prenatal antibiotics (196)
Asthma	*Veillonella* (256) *Christensenellaceae* spp. (200)	*Roseburia, Alistipes, Faecalibacterium, Bifidobacterium, Ruminococcus, Dialister, Lachnospiraceae incertae sedis* (54, 256).	↓ Butyrate, propionate (204) ↓ Polyunsaturated fatty acids (200) ↑ *γ*−/β-tocopherol (200)	Positive factor: PA (202, 203) Negative factor: Early-life antibiotics (139) Prenatal antibiotics (196) Western diet (200)
Allergic rhinitis	*Actinomyces, Bacteroides* (174)	*Bifidobacterium* and *Escherichia/Shigella* (174)	↓ Butyrate (204)	Positive factor: Postbiotics (204) Synbiotics (174) Negative factor: Prenatal antibiotics (196)
Food allergies	*Salmonella*, *Enterobacter*, *Bacteroides*, *Trabulsiella* (257) *Ruminococcus*, *Lactococcus* (258)	*Clostridia*, *Firmicutes* (257) *Leuconostoc* (258)	↑ Plasma ornithine, bilirubin, taurocholate, carnitine, hypotaurine ↓ α-ketobutyrate (259)	Positive factor: Postbiotics (204)
Neurodevelopmental disorders				
Attention-deficit/hyperactivity disorder (ADHD)	*Collinsella*, *Akkermansia*, *Blautia*, and *Dorea* (51)	*Lachnospiraceae*, *Enterococcus*, *Ruminococcus*, *Lactobacillales* (51) *Bifidobacterium* (233)	↓ GABA, lactic acid (51) ↑ 3-methylazelaic acid (260) ↓ Dopamine 4-sulfate, citrulline, propanoate (260)	Positive factor: Psychobiotics (230) Prenatal healthy diets (232) Negative factor: Prenatal western diet (232) Tobacco exposure (229)
Autism spectrum disorders (ASD)	*Clostridium*, *Klebsiella*, *Blautia*, *Ruminococcus*, *Coprobacillus* (186) *Faecalibacterium prausnitzii* (261)	*Bifidobacterium*, *Alistipes*, *Parabacteroides* (186)	↓ Fecal GABA ↑ Tryptophan and isoleucine (186). ↑ Butyrate (186, 261)	Positive factor: Vaginal microbiota transfer (61) Maternal SGS diet (106) PA (231) Negative factor: Antenatal infections (229)
Gastrointestinal diseases				
Inflammatory bowel diseases (IBD)	*E. coli*, *Prevotella*, *Bacteroides*, *Enterococcus* (175)	*Bifidobacterium longum*, *Bifidobacterium pseudocatenulatum*, *Eubacterium*, and *Clostridium* (175)	↑ Fecal calprotectin (175) ↓ Tryptophan, succinate and 3-hydroxyisobutyrate (179)	Positive factor: Phage therapy (148) Breastfeeding (206) Early postnatal fiber diet (213) Negative factor: High sugar-sweetened beverages (213) High-fat diet (211)
Irritable bowel syndrome (IBS)	*Flavonifractor plautii*, *Lachnospiraceae bacterium 7_1_58FAA*, *Oscillibacte*r (178)	*Bifidobacterium* and *Eubacterium* (176)	↑ Glucose, hyocholate, sterols, and lactate (176, 178) ↓ Butyrate, thymine (176, 178)	Positive factor: Psyllium supplements (214) Breastfeeding (208) Negative factor: FODMAPs (176) Early-life stress (207)
Metabolic disorders				
Type 1 Diabetes Mellitus (T1DM)	*Bacteroides dorei* and *Bacteroides vulgatus* (192)	*Coprococcus* (192)	↓ Glutamate, 4-hydroxyphenyllactic acid, ribonic, tryptophan, aspartate, succinate (262). ↑ Methionine (262)	Negative factor: Prenatal antibiotics (216) Positive factor: Breastfeeding (218) Probiotics (222)
Childhood obesity	*Faecalibacterium*, *Tyzzerella*, *Klebsiella* (263) *Streptococcus* (264) *Pseudobutyrivibrio* and *Lactobacillus* (193)	*Bifidobacterium longum, Collinsella, F. prausnitzii* (264) *Clostridium sensu stricto 1* and *Akkermansia* (193)	↓ Serum GABA, symmetric dimethylarginine and asymmetric dimethylarginine (193) ↑ Glutamic acid, acetylcarnitine, carnitine and threonine (193)	Positive factor: Phytonutrients (103, 104, 168) PA (126) Breastfeeding (219) Maternal probiotics (223) Negative factor: Early-life antibiotics (139) Intrauterine antibiotics (217)

↑ increase. ↓ decrease. FODMAPs, fermentable oligosaccharides, disaccharides, monosaccharides and polyols.

### 4.1 Allergic conditions

Pediatric allergic diseases, including eczema, asthma, allergic rhinitis, and food allergies, are common chronic conditions characterized by immune hypersensitivity and often share a genetic and environmental basis. Infant eczema, also known as atopic dermatitis (AD), is the most common inflammatory skin disease affecting up to 15–30% of children (37, 169). Asthma is an inflammatory disease affecting the respiratory system in about 10% of the pediatric population (170). Allergic rhinitis (AR) is characterized by distinct and easily identified symptoms such as persistent sneezing, itching, as well as nasal discharge and congestion, affecting about 2–25% of children. The prevalence of childhood food allergies has increased in recent years, with an estimate of about 10%, particularly in developed countries (171). AD, asthma, AR and food allergies are common childhood atopic diseases, regulated by exaggerated IgE immune response to otherwise harmless substances in the environment. Research has demonstrated that children with AD or asthma exhibit increased susceptibility to at least one additional form of atopy (169, 170).

Studies have suggested that the gut microflora acts as an important regulator in atopic pathogenesis. Childhood gut dysbiosis, characterized by lower *α* and *β*-diversities, delayed gut microbiota maturation and reduced SCFAs-producing bacteria, has been associated with the development of pediatric atopic diseases (171–173). However, it is important to mention that certain microbiota associated with food allergies in young children differ depending on the food allergens (174). Given that the gut microbiota influences the development and maturity of host immune system early in life, it has been hypothesized that decreased SCFA butyrate concentration could lead to suppressed differentiation of naïve T-cells into Tregs, which consequently deduces the ability of Tregs to suppress excessive immune responses. Accompanied by gut microbial imbalance, fewer mucosal IgA-producing plasma cells and elevated serum IgE levels mediated by Th2-type cells are mostly observed in pediatric patients with allergic diseases (169, 173). Table 1 summarized the changes of the gut microflora and metabolites in common pediatric allergic diseases.

### 4.2 GI diseases

IBD and IBS are common GI disorders among the pediatric population (175, 176). IBD defined as an autoimmune and chronic inflammatory condition of the intestines, primarily including Crohn’s disease (CD) and ulcerative colitis (UC) (175, 177). IBS, a chronic medical condition, consists of a group of symptoms that co-occur, including abdominal pain and abnormal bowel movement (176). Although the association between the early-life gut bacterial composition and the etiology of IBS and IBD remains unclear, studies have found that IBD and IBS in the older children are associated with altered bacterial composition (176, 178) (Table 1). Children with IBS have commonly shown a high ratio of class *Gammaproteobacteria*, and reduced genera *Bifidobacterium* and *Eubacterium* (176). Depending on IBD categories, certain bacterial changes have also been reported in pediatric patients with UC and CD (175) (Table 1). Correspondingly, pediatric patients with IBD have lower amounts of key microbial metabolites such as tryptophan, succinate and 3-hydroxyisobutyrate (179). Also, increased glucose, sterols and lactate, as well as reduced butyrate have been found in kids with IBS (176). Nevertheless, the early life metabolomic profiles associated with pediatric IBS and IBD are still under-researched.

For the immunopathogenesis of IBD and IBS, dysregulated mucosal immune response caused by epithelial barrier defects and microbial dysbiosis is the main driving factor. For instance, certain mucus-degrading pathobionts, such as adherent-invasive *Escherichia coli*, can induce IL-1β secretion from mononuclear phagocytes such as macrophages, promoting the differentiation of Th17 cells and gut inflammation. Also, colonization by oral-derived *Klebsiella* species promotes the production of proinflammatory cytokines from DCs and macrophages, which facilitate the differentiation of naïve T cells into Th1 and Th17 cells (56). Intestinal recruitment of plasma cells with high concentration of IgG, has been involved in the progression of IBD (180, 181). Likewise, increased innate immune activity, particularly from mast cells and monocytes, in the colonic mucosa and the blood, has been observed in IBS. In cohorts with IBS, adaptive immune response is observed in affected children by increased numbers of T-cells, altered mucosal B-cells activity, and antibody production in the intestine (182, 183). However, these relevant mechanisms require further validation in young children with elevated risks of IBD and IBS.

### 4.3 Neurodevelopmental diseases

The critical developmental period involved in microbial plasticity and variability coincides with neurodevelopmental stages, including synaptogenesis and myelination of the CNS during the first few years of life (7, 24, 51). The gut-brain axis also elucidates the impacts of the gut microbiome on the pathogenesis of common neurodevelopmental conditions, such as ASD and ADHD (52, 184). ASD is a complex neurodevelopmental disorder that greatly affects a child’s communication and social ability (184). ADHD, a chronic developmental condition associated with hyperactivity, inattention and impulsivity, begins in childhood and can extend into teenage and adulthood (51, 185). ADHD can occur in children diagnosed with ASD, and vice versa (184).

As mentioned previously, the enteric microbes influence neurological development and functions via microbial-derived metabolites and immune pathways. Thus, the gut microbiota is considered as a potential contributor to ASD and ADHD pathogenesis (51, 52). Notably, in young children with high risk of ASD and ADHD, studies have reported different gut microflora profiles and bioactive metabolic products as compared to normal controls. For instance, a low amount of bacterial-derived neuroactive molecules such as GABA has been observed in young children at risk of ASD and ADHD (Table 1). This metabolic shift is positively associated with depleted *Bifidobacterium* spp. and *Lactobacillus* spp., as well as abundance of *Clostridium* in high-risk children (51, 186). These findings further indicate the significant connection between the gut microbiota-metabolic interface and neurological conditions during early life. Furthermore, impaired inflammatory responses and plasma cytokine levels are observed in young pediatric patients with ASD (187). Of note, GABA is not only a major inhibitory NT, but also an immunomodulatory molecule that can indirectly influence the brain functioning through the gut-brain axis. Altered levels of GABA and cytokines found in ASD and ADHD cohorts indicate a systemic inflammation in these neurological disorders, subsequently leading to a gradual disruption of the blood–brain barrier and neuroinflammation (34, 51, 186). However, further studies are needed to reveal the early-life immunological signatures in the onset of ASD and ADHD during the critical window of microbial development.

### 4.4 Metabolic diseases

Pediatric obesity and T1DM are the most common metabolic diseases associated with early-life gut dysbiosis. Over the past years, obesity has become a global epidemic not only in adults but also in children (188, 189). Childhood obesity is classified as a body mass index (BMI) at or above the 95^th^ percentile for a child’s age and sex (189). T1DM is an autoimmune disease caused by an irreversible destruction of pancreatic islet *β*-cells, leading to insulin deficiency. TIDM is prevalent in the younger age group compared to adults. Of note, the increased prevalence of childhood obesity has been shown to positively associate with the increased risk of T1DM among children (188, 190). It is widely reported that both pediatric obesity and T1DM are associated with imbalance of the early-life gut microbiota. Importantly, the early-life gut microbiota plays crucial roles in host metabolic pathway, including lipid and glucose metabolism, energy expenditure and fat storage, and insulin signaling (13, 188, 191). T1DM usually develops within the first 5–6 years of life, which coincides with the crucial stage of gut microbial development (192). Compared to healthy controls, the abundances of *Bacteroides dorei* and *B. vulgatus* have been found significantly increased in young children at high risk of T1DM autoimmunity prior to seroconversion (Table 1). Notably, these *Bacteroides* spp. are linked to gut inflammation (192). Similarly, children with obesity have exhibited higher abundance of *Bacteroides fragilis*, and reduced *Bifidobacteria* and *Collinsella* from their early infancy, which are associated with excessive weight gain (13).

Early-life metabolic dysregulation may precede the pathogenesis of these pediatric metabolic diseases. For instance, children with overweight and obesity are shown to have significantly lower amounts of GABA, which regulates food intake and body weight. It is concluded that symmetric dimethylarginine and asymmetric dimethylarginine observed in children with obesity may be associated with systemic inflammation (193). Another study by Azab et al. observed that circulating metabolites involved in gluconeogenesis, amino acid and fatty acids metabolisms are significantly increased in 5-year-old children with metabolic syndrome. These metabolic pathways were strongly associated with increased BMI-for-age, waist circumference and fasting blood glucose in the pediatric cohorts (194).

The interplay between the early gut microbiome and immunity in children with obesity and T1DM has recently received significant research interest. Increased C-reactive protein has been observed in severe obese children between ages 3–5 years (195). Altered bacterial composition and catabolites may impair adipose tissue homeostasis and induce inflammation contributing to childhood obesity (13, 189). Furthermore, pediatric T1DM patients often have co-occurred gut dysbiosis as well as elevated systemic and tissue-specific inflammatory responses, indicating key roles of gut microbes in maintaining host metabolic health (188, 190).

## 5 Manipulating the early-life gut microbiome in common pediatric diseases

Reshaping the early-life gut microbiota through microbial-based interventions is an effective approach to promote overall health throughout childhood to adulthood. The gut microbiota at childhood stage is more susceptible to environmental factors than adulthood. Moreover, the critical life stages in infancy and early childhood coincide with the maturation of the immune system, which is also regulated by the gut microbiota. Thus, microbial manipulation during early life may deliver long-term health benefits (20). Although microbiota-derived intervention has been widely applied in prevention and treatment of various human diseases, research on microbial manipulation targeting pediatric diseases remains underexplored. Herein, we review the advanced studies involved in manipulating the early-life gut microbiome in common childhood diseases outlined in Table 1.

### 5.1 Allergic diseases

Numerous evidence has reported that prenatal and postnatal exposure to antibiotics is linked to an increased risk of allergic diseases in children. For instance, a Taiwan population-based study demonstrated that prenatal exposure to antibiotics increased the risk of pediatric allergic diseases in a dose-dependent manner (196). Another study found that macrolide use early in life is associated with a disrupted microbiome, including depleted phylum *Actinobacteria*, and increased phyla *Bacteroidetes* and *Proteobacteria* (139). Microbial shift due to antibiotics use is also associated with increased risk of asthma and high BMI.

Interestingly, a recent study by Chan et al. investigated the efficacy of a novel infant formula (SIM03) containing viable *Bifidobacterium bifidum* and *Bifidobacterium breve* strains, in preschool children with eczema (197). The result showed a significant abundance of *B. breve* and microbial enrichment involved in acetate and acetyl-CoA synthesis, which is correlated with symptom relief of eczema. In a Finnish study, four probiotic strains with galacto-oligosaccharides were administered to mothers at 36th week of gestation and to their infants until 6 months of age (174). In the rhinitis group, probiotics intervention promoted the significant abundance of *Bifidobacterium* and reduced *Bacteroides*. Another similar study by Kukkonen et al. demonstrated that probiotics and galacto-oligosaccharides administration enriched the bacterial species *Lactobacilli* and *Bifidobacteria*, leading to eczema prevention (198).

Studies have shown that breastfeeding plays a central role in developing a tolerogenic immune response for infants. However, evidence on associations between breastfeeding and allergic diseases seems inconsistent. Some studies report a protective effect (199, 200), while others reported no preventive effect of prolonged breastfeeding (199, 201). With respect to early childhood exercise or PA during the plastic phase of the microbiome, a cohort study by Byberg et al. showed that low PA at 3–6 years was positively associated with asthma in late childhood (202). Similarly, Firrincieli et al. observed a significant association between decreased levels of PA and history of asthma or wheezing among preschool children (203). However, the gut microbial variation was not analyzed in both studies.

Early-life nutrition and dietary habits are crucial factors influencing gut microbiota development and the onset of pediatric atopic diseases. According to a PASTURE study, infants with high fecal levels of butyrate and propionate at 1 year of age had a decreased risk of developing asthma and atopic sensitization (204). Fecal SCFAs showed a significant association with infants’ dietary intake of yogurt, fish, fruits and vegetables. Young children with high levels of butyrate are less likely to develop food allergies and AR, indicating the use of SCFAs could be protective against pediatric atopy diseases. Besides, infants with ≥3 siblings showed high propionate levels (204). Another metabolomic study indicates a positive association between diet rich in fried and processed meat and asthma as well as the increased abundance of *Christensenellaceae* (200). Nevertheless, the effectiveness of these approaches targeting the early-life microbiome against the onset of allergic diseases in children requires further validation.

### 5.2 GI diseases

Mounting evidence has uncovered the roles of early-life antibiotic exposure in childhood GI diseases. A systemic review of 22 studies discovered a strong association between antibiotics use in the first 2 years of life and the risk of IBD later in life (205). Similarly, Agrawal et al. in a systemic review and meta-analyses discovered that prenatal exposure to antibiotics and tobacco smoke were positively associated with offspring IBD (206). On the other hand, phage therapy that shows safety and tolerability in humans could be a treatment approach in pediatric GI diseases (148). However, more extensive research is still needed to understand the relationship between early-life antibiotics use, gut microbiota, and the onset of IBS and IBD.

Notably, early-life stress has shown to play an exacerbating role in pediatric GI disorders. A recent animal study reported that early-life stress using the maternal separation model induced IBS from childhood to adulthood in mice (207). Intestinal bacterial shift showed decreased *Lactobacillus*, *Enterorhabdus, Ruminococcus* and *Dubosiella*. Despite the encouraging data in animals, the effect of breastfeeding to enhance resilience against IBD or IBS remains controversial in humans (206, 208, 209). Over the past years, early-life intake of probiotics in infants has shown promising health benefits (210). Although the use of prebiotics, synbiotics, postbiotics, and para-probiotics early in life could be promising tools for treatment and prevention of pediatric GI conditions, they have not been applied as a standard IBD treatment. More well-designed randomized-controlled trials are required to better understand the intricate interplay between the role of these approaches and early-life gut microbiota in pediatric GI diseases.

Maternal and early postnatal dietary intervention are promising strategies for prevention of GI diseases in children. For instance, a high-fat diet and diets rich in fermentable oligosaccharides, disaccharides, monosaccharides and polyols have been shown to exacerbate the symptoms of IBD and IBS (176, 211). Polyphenols, minerals and high fiber diets have been reported to alleviate the symptoms of IBS and IBD (212). Data from a pooled study in two Scandinavian birth cohorts observed that high intake of fish and vegetables at 1 year old was associated with a reduced risk of IBD, while a high intake of sugar-sweetened beverages was associated with an increased risk of IBD (213). Intriguingly, herb use such as psyllium supplementation could be beneficial for pediatric IBS (214). According to a randomized double-blind trial of 103 children with IBS, children fed psyllium fiber for 6 weeks had less abdominal pain episodes than children in the placebo group (215). However, there were no differences regarding the microbial composition between the two groups.

### 5.3 Metabolic diseases

Maternal and early childhood exposure to antibiotics is closely associated with high risk of childhood metabolic diseases. A study in Finland population found that maternal use of phenoxymethyl penicillins or quinolone antimicrobials before pregnancy was associated with an increased risk of T1DM in children (216). Also, maternal macrolides used before pregnancy were associated with a significant diabetes risk in children. Another finding by Zhou et al. showed that intrauterine antibiotic exposure was associated with infant growth retardation (217). In this study, intrauterine penicillin was positively associated with low diversity and relative abundances of *Proteobacteria*, *Bacteroidetes*, and *Gammaproteobacteria,* but negatively associated with the presence of *Firmicutes, Lactobacillales*, *Bacillales*, and *Staphylococcaceae* in infants.

During the first months of life, HBM serves as the primary nutrition source, shaping the early-life intestinal microbiota and promoting a *Bifidobacterium*-dominated gut. Data from two population-based cohorts revealed that the risk of T1DM doubled in those who were not breastfed (218). Among infants who were breastfed, no significant difference was observed upon comparing the duration of breastfeeding. In a meta-analysis of 25 studies, Yan et al. revealed that breastfeeding is a significant protective factor against childhood obesity (219). However, most of these studies fail to establish a potential correlation between the early-life microbial changes and dysmetabolism.

The use of prebiotics, probiotics and synbiotics has shown a positive effect on improving gut health and reducing the risk of metabolic diseases (220, 221). Studies by Uusitalo et al. showed that early probiotic intervention during the first 27 days of life was associated with a reduced risk of islet autoimmunity among children at risk for T1DM (222). According to studies by Saros et al., probiotics administration of *Lactobacillus rhamnosus HN001* and *Bifidobacterium animalis ssp*. *lactis 420* alone or combined with fish oil during pregnancy and the first 6 months postpartum decreased the risk of excessive adiposity in the infants at 24 months of age, suggesting that maternal probiotics intervention may prevent childhood obesity (223). However, studies examining the effectiveness of probiotics and synbiotics in preventing T1DM and childhood obesity during early life are scarce. The use of postbiotics and para-probiotics has been proposed to alleviate metabolic and gut microbial disturbances through multiple mechanisms (122, 224). Moreover, microbiota engraftment or FMT have been suggested to offer potential preventive and therapeutic options for children at risk of metabolic diseases. However, most human studies highlight the beneficial role of microbiota transplants in adults (225, 226). Despite their favorable effects, microbiota transplants remain underutilized in the pediatric population for preventing or treating childhood metabolic diseases.

PA is considered a major modulator for gut microbiota. In pediatrics with metabolic diseases, studies have reported the impacts of PA in combatting adverse metabolic effects and reversing gut microbial aberrations. In children with obesity, studies have shown that PA reduced plasma glucose levels and pro-inflammatory pathways with lowered *gammaproteobacteria*, and increased *Roseburia*, *Blautia* and *Veillonella* (126). Metabolic profiling revealed an increase in SCFAs after exercise intervention. However, these studies were done in older children or adolescents when the gut microbiota has attained an adult-like characteristic. It is difficult to link PA impacts to early-life microbiota status in children at infancy or early childhood stages. Further research is urgently needed to address the impacts of PA on the highly plastic gut microbiome in infantile and early childhood as well as their metabolic outcomes.

Early diets and nutrients, including gluten, fat, fiber, and micronutrients such as vitamins D and E, selenium, and zinc, are implicated in metabolic regulatory roles on influencing gut microbial composition, energy metabolism, and pancreatic function during early life (220, 227). Studies have shown that maternal microbial status, influenced by specific dietary intake during pregnancy, can reshape the early-life gut microbiome, thereby contributing to the infant’s metabolic development (228). Beneficial phytochemicals such as polyphenols are known to exhibit prebiotic potential and decrease the risk of metabolic syndromes (102, 103). In an animal study, grape polyphenol intake early in life stimulated the colonization of *Akkermansia* and *Lactobacillu*s, SCFAs release, and key genes involved in mucosal integrity (103). Also, studies from our lab have shown that early-life broccoli glucoraphanin (168) or maternal soy genistein treatment (104) can reduce excessive adiposity and improve overall metabolic health in offspring from early childhood, leading to prevention of later-life chronic diseases in adulthood through regulation of the gut microbiome. Further translational studies are needed to understand how these nutritional components influence the early-life gut microbiome in pediatric metabolic diseases.

### 5.4 Neurodevelopmental diseases

A recent population-based cohort study on a total of 66 Swedish babies indicates that longer exclusive breastfeeding reduced the risk of ASD and ADHD (229). On the contrary, maternal smoking, tobacco exposure, stress and short breastfeeding duration (< 4 months) are significantly associated with increased risk of ADHD. Infections during pregnancy and maternal autoimmune diseases are positively correlated with ASD development. In addition, Zhou et al. demonstrated vaginal microbiota transfer (VMT) to newborns may reverse C-section-related microbiome disturbances (61). For example, infant neurodevelopmental scores measured at 6 months showed significant improvement in VMT group compared to control. Microbial and metabolomics dynamics in VMT group showed significant enriched presence of *Lactobacillus*, *Bifidobacterium*, *Escherichia*, and reduced *Klebsiella*, as well as elevated L-lactic acid, acetate and indolelactic acid. Probiotic intervention (psychobiotics), contributing to reduced risk of neuropsychiatric disorder in early childhood. In a study by Pärtty et al., 75 infants received *Lactobacillus rhamnosus GG* supplement during the first 6 months of life showed increased fecal *Bifidobacterium* at 6 months, *Bacteroides* and *Lactobacillus-Enterococcus* at 18 months, and *Clostridium histolyticum* group at 24 months, leading to reduced risk of ADHD by the age of 13 years old (230). Furthermore, PA, especially aerobic exercise prior to classroom activities, has been demonstrated to significantly improve academic responding in young children with ASD (231). However, the gut microbiota variations were not reported in this study.

Maternal gut microbiota influenced by the dietary pattern has been shown to impact brain function in the offspring. The *Barwon Infant* Cohort Study (232) discovered that higher maternal *α*-diversity was associated with better behavioral outcomes at 2 years of age in the offspring. Butyrate producers from the *Lachnospiraceae* and *Ruminococcaceae* families were more abundant in mothers of the children in the normal behavioral group. A healthy prenatal diet is positively associated with increased maternal microbiota α-diversity and reduced internalizing behaviors in children, suggesting the roles of maternal diet in support of early-life brain development in infants. In addition, studies have reported that omega-3 fatty acids, vitamins, zinc, magnesium and phytochemicals may exert beneficial effects in managing dysbiosis associated with ASD and ADHD (233).

### 5.5 Other common pediatric diseases

In the pediatric population, emerging studies have reported that gut microbiome is implicated in common pediatric endocrinological diseases such as thyroid dysfunctions, affecting about 3.7% of the U.S. children (234). Gut dysbiosis and intestinal permeability have been linked to increased risk of autoimmune thyroid disease (AITD), particularly Hashimoto’s thyroiditis and Graves’ disease, which are major causes of hypothyroidism or hyperthyroidism. Studies have provided supporting evidence of the gut-thyroid axis, which is considered a complex interrelationship between the gut microbiota and thyroid gland (235). Enteric bacteria can influence the bioavailability of minerals, including copper, iodine, iron, selenium and zinc as well as hormone-metabolizing enzymes that support thyroid hormone synthesis and metabolism (235, 236). On the other hand, thyroid hormones impact GI motility and potentially mediate gut microbial composition (237–239). Thyroid immunity is also regulated by the gut microbes and their metabolites, thus contributing to the pathogenesis of AITD (236). While studies in the adult population have shown significant decreased *Bifidobacteria* and *Lactobacilli* in patients with hyperthyroidism (238), and enriched *Akkermansia*, *Bifidobacterium* and *Lachnospiraceae* in hashimoto’s thyroiditis subjects (239), research in children, especially during the critical window of microbial development, remain largely underexplored. Also, studies have reported that intake of high fiber diet, thyroid-related micronutrients, probiotics and prebiotics interventions (235, 236, 240), physical activity (241), and FMT (242) may have protective roles on thyroid functionality and overall gut health, whereas endocrine disruptors contribute to dysbiosis-associated AITD (243). Further research is warranted regarding how this interaction influences thyroid dysfunctions in pediatric group.

Precocious puberty (PP) is a prevalent endocrine disorder in pediatrics, characterized by early maturation of sexual characteristics and gonadal function in children before ages 8 and 9 for boys and girls, respectively. PP is more common in girls than in boys. Depending on activation of the hypothalamic–pituitary-gonadal-axis, PP is categorized into central and peripheral precocious puberty (244–246). Through the sex hormone-gut microbiome axis, gut microbiota influences the development of secondary sex characteristics and the onset of puberty. For instance, *Ruminococcus*, *Faecalibacterium*, and *Bacteroides* species are known to impact estrogen metabolism, particularly through *β*-glucuronidase activity (247, 248). Gut microbial-derived SCFAs and NTs have also been reported to mediate puberty timing via the gut-brain axis (246, 248). In recent years, increasing evidence has shown that gut dysbiosis contributes to the onset of PP (246, 247). A cross-sectional study conducted by Dong et al. showed increased *R. gnavus*, *Ruminococcus callidus*, *Ruminococcus bromii*, *Roseburia inulinivorans*, *Coprococcus eutactus*, *Clostridium leptum*, and *Clostridium lactatifermentans*, in preadolescent girls with central PP compared to healthy controls. These enriched taxa are also associated with obesity, SCFAs production and estrogen metabolism (245). Fecal metabolome signatures of girls with PP showed significant increase in β-glycerophosphoric and hexacosanoic acids that are positively associated with luteinizing and follicle-stimulating hormones secretion. Moreover, metabolomics enrichment showed elevated obesogenic and PP associated pathways, including steroid biosynthesis, ovarian steroidogenesis and glycerophospholipid metabolism (244). Despite these significant findings, the early-life gut microbiota and metabolome of young children at risk of early onset puberty has not yet been fully elucidated.

Regarding microbial remodeling in early pubertal onset, nutritional interventions have been found to be highly important. For instance, vitamin D supplementation and probiotics have been suggested as promising therapeutics for PP in children, but larger studies are warranted to determine effective dosages (249). Similarly, animal experimental studies have demonstrated that probiotics treatment could reverse early onset of puberty induced by early-life stress and soy daidzein (250, 251). According to Wang et al., complex carbohydrate and high-protein diets may have protective effects against PP in females. The consumption of nuts, vegetables, seafood, freshwater products, and livestock and poultry meat may beneficially contribute to the reduced risk of PP (252). Emerging evidence also indicates that early-life exposure to endocrine-disrupting chemicals and environmental pollutants can alter the gut microbiome, thus increasing the risk of PP (243, 253). Nevertheless, further research is needed to fully elucidate the underlying mechanisms. Furthermore, more studies are required to investigate the gut microbes and their manipulating factors in influencing the risk of early onset puberty in critical microbial development window.

## 6 Limitations

This narrative review did not adhere to a systematic literature search and screening protocol, such as PRISMA guidelines. Therefore, the selection of included studies may be subject to potential selection bias, reflecting the authors’ perspectives or omitting some relevant literatures. Based on scientific evidence, majority of the research linking the early-life microbiome to pediatric diseases relies on observational studies, making it difficult to establish definitive causality. There are significant variabilities across the discussed studies, including study designs, participant characteristics, microbiome analysis, and reported clinical outcomes, thus complicating direct comparisons and synthesis. Longitudinal studies tracking children from birth for disease development are still relatively limited for some pediatric conditions. Mechanistic studies, particularly in humans, are needed to fully elucidate the causal pathways. Regarding the interactions between the host, the gut and diseases, studies mostly rely on fecal samples to characterize the gut microbiome, which may not fully represent microbial activity along the entire GI tract.

## 7 Conclusion and future directions

**Figure 4:**
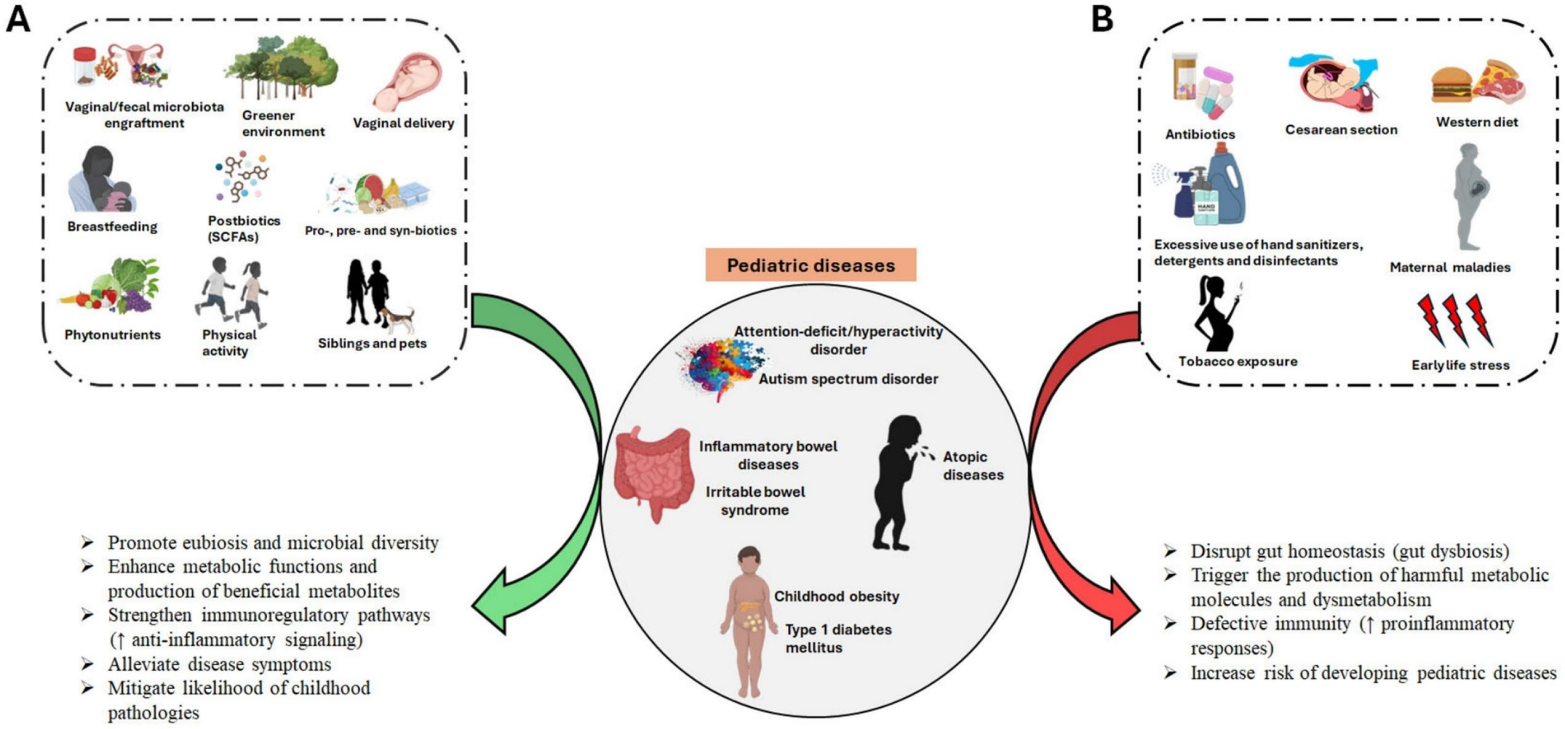
Influence of microbe-based modifiable factors on childhood disease development. **(A)** Positive modulators. **(B)** Detrimental modulators. Created with BioRender.com.

Imbalanced microbial profiles and the subsequent disturbances in metabolites and immune responses are frequently observed in pediatric diseases. The early-life gut microbiome is more dynamic and sensitive to the environmental factors compared to adulthood. Hence, during this crucial period, the gut microbiome is highly susceptible to external influences, which can significantly impact early development and health outcomes in children. Modulating the gut microbiome at early age is an effective strategy in prevention of childhood diseases through dietary and non-dietary intervention approaches. This highlights the importance of microbiome-targeted strategies in fostering a healthy gut and protecting children from dysbiosis-related diseases throughout life. Strengthening the early-life gut microbiome presents a promising therapeutic approach to enhancing metabolomic profiles and immunological signatures from early life. Notably, adhesion to breastfeeding, biotics, physical activity, and a healthy diet may help reduce the risk of these diseases from early childhood into adulthood (Figure 4).

Despite significant advancements in understanding the human gut microbiome, research on the interaction between early-life gut dysbiosis and pediatric diseases remains limited. The early-life gut microbiome plays a crucial role in disease onset, yet most clinical evidence focuses on older children and adults, whose gut microbiota stabilizes after ages 3 and 6. Therefore, more translational studies are needed to clarify how early-life gut microbes and their metabolites influence both early and later disease development. Additionally, further research should explore intervention strategies to counteract dysbiosis-related diseases in the pediatric population and promote a healthy intestinal microbiome from early lifetime.

## Glossary

AD: Atopic dermatitis
ADHD: Attention-deficit/hyperactivity disorder
AMPs: Antimicrobial peptides
AR: Allergic rhinitis
ASD: Autism spectrum disorder
AT: Adipose-tissue
AITD: Autoimmune thyroid disease
BEVs: Bacterial extracellular vesicles
BMI: Body mass index
CD: Crohn’s disease
CNS: Central nervous system
COVID-19: Coronavirus disease 2019
CS: Cesarean section
DCs: Dendritic cells
ENS: Enteric nervous system
FMT: Fecal microbiota transplantation
GABA: *γ*-aminobutyric acid
GI: Gastrointestinal
GPCRs: G-protein coupled receptors
HBM: Human breast milk
HMOs: Human milk oligosaccharides
HPA: Hypothalamic–pituitary–adrenal
IBD: Inflammatory bowel diseases
IBS: Irritable bowel syndrome
NTs: Neurotransmitters
PA: Physical activity
PP: Precocious puberty
Tregs: Regulatory T cells
SCFAs: Short-chain fatty acids
SGS: Sulforaphane glucosinolate
T1DM: Type 1 diabetes mellitus
Th: T-helper
TLR: Toll-like receptors
UC: Ulcerative colitis
VMT: Vaginal microbiota transfer
WHO: World Health Organization
